# Implementation quality as a modifiable determinant of filter lifespan in regional citrate anticoagulation: a real-world clustering-adjusted study

**DOI:** 10.1080/0886022X.2026.2656550

**Published:** 2026-04-22

**Authors:** Qing Ou, Wenjuan Yan, Zhili Liang, Yanan Liu, Hongbin Hu, Hongjuan Wang, Dan He, Yucheng Li, Hongyun Wei, Zhenhua Zeng

**Affiliations:** Department of Critical Care Medicine, Nanfang Hospital, Southern Medical University, Guangzhou, China

**Keywords:** Continuous renal replacement therapy, regional citrate anticoagulation, heparin, nafamostat mesilate, filter lifespan, cluster analysis

## Abstract

Continuous renal replacement therapy (CRRT) is widely used in critically ill patients, but filter clotting remains a common complication. Regional citrate anticoagulation (RCA) is recommended; however, its real-world effectiveness may depend on implementation quality. This single-center retrospective cohort study analyzed 420 CRRT sessions from 1 September 2025 to 30 November 2025. Mixed-effects Cox models with patient-level random intercepts and competing-risk analysis were used. The primary outcome was filter clotting. In adjusted analyses, RCA was associated with a significantly reduced hazard of filter clotting compared with heparin (adjusted hazard ratio (aHR) for heparin vs. RCA: 1.78, 95% confidence interval (CI): 1.14–2.77, *p* = 0.011). Nafamostat showed no statistically significant difference from RCA (aHR: 1.31, 95% CI: 0.78–2.19, *p* = 0.31); however, the wide CI reflects limited sample size, and equivalence cannot be inferred. Within the RCA group, absence of timely post-filter ionized calcium (iCa^2+^) monitoring within 2 h was independently associated with increased clotting risk (adjusted odds ratio: 2.18, 95% CI: 1.24–3.84, *p* = 0.007), with a dose–response relationship (each one-hour delay increased clotting odds by 15%, *p* = 0.02). Major bleeding was infrequent (2.1% overall); metabolic complications (citrate accumulation) occurred in 2.9% of RCA sessions. In this cluster-adjusted real-world cohort, RCA was associated with improved filter survival compared with heparin. Implementation fidelity, particularly timely post-filter iCa^2+^ monitoring, appears to influence RCA effectiveness.

## 1. Introduction

Continuous renal replacement therapy (CRRT) is a key component of critical care for patients with acute kidney injury, fluid overload, or severe electrolyte disturbances [1]. Effective anticoagulation of the extracorporeal circuit is essential to maintain patency and ensure treatment efficacy [2].

Regional citrate anticoagulation (RCA) has emerged as a preferred strategy due to its localized effect, which reduces the risk of systemic anticoagulation and bleeding [3,4]. Current guidelines recommend RCA for CRRT in patients without contraindications [5]. However, RCA management is complex, requiring vigilant monitoring of post-filter and systemic ionized calcium (iCa^2+^) levels to balance efficacy against the risk of citrate accumulation [6]. This complexity can inhibit its consistent application in routine practice.

Systemic unfractionated heparin remains extensively used due to familiarity and low cost; however, it carries risks of bleeding and heparin-induced thrombocytopenia (HIT) [7]. Nafamostat mesilate, a short-acting, broad-spectrum serine protease inhibitor with a favorable bleeding profile, is available and used in certain Asian countries; however, the comparative evidence is limited [8,9].

The meta-analyses of randomized trials suggest that RCA improves circuit lifespan compared with heparin [10]; however, the data from real-world and less-controlled settings are limited. Furthermore, direct comparisons involving nafamostat are limited [11]. Notably, the majority of real-world studies do not consider the clustering introduced by repeated CRRT sessions within the same patient, potentially violating statistical independence assumptions and inflating the precision of estimates.

Therefore, this large, real-world cohort study aimed to comprehensively compare the effectiveness of RCA, heparin, and nafamostat for CRRT using rigorous statistical methods that account for patient-level clustering and competing risks. We hypothesized that the clinical superiority of RCA is dependent upon rigorous monitoring practices and that failure to account for within-patient correlation can overestimate treatment effects.

## 2. Methods

### 2.1. Study design and population

This single-center, retrospective cohort study reviewed all CRRT sessions performed in a university-affiliated intensive care unit (ICU) from 1 September 2025 to 30 November 2025. The study was approved by the Institutional Review Board (Protocol NFEC-202505-K34) with a waiver of informed consent.

The inclusion criteria were as follows: (1) age ≥ 18 years; (2) CRRT (continuous venovenous hemofiltration (CVVH), continuous venovenous hemodialysis (CVVHD), or continuous venovenous hemodiafiltration (CVVHDF)) duration > 4 h.

*Statistical unit clarification*: Initially, each CRRT session was considered as an observational unit; however, multiple sessions could originate from the same patient. All regression analyses included patient-level clustering through the use of robust variance estimation or mixed-effects modeling to account for within-patient correlation and prevent the violation of independence assumptions, as specified below.

### 2.2. Data collection and variables

Data were extracted from electronic medical records and dedicated CRRT flow sheets into a standardized database. Key variables included:

*Demographics*: Age and sex.

*Treatment parameters*: CRRT mode, machine type (Fresenius multiFiltrate (Fresenius Medical Care, Bad Homburg, Germany) or Baxter Prismaflex (Baxter International, Deerfield, IL)), blood flow rate, dilution mode (pre-filter anticoagulant infusion), total treatment time, dialysate flow rate, replacement fluid rate, CRRT dose, time from ICU admission to CRRT start, and CRRT indication. Blood flow rate was set at 150–180 mL/min based on the pathophysiological characteristics of critically ill patients (e.g., sepsis, hypercatabolic states, and severe fluid overload) to achieve an adequate CRRT dose of 25–30 mL/kg/h while maintaining citrate infusion within safe limits. This approach reduces filtration fraction and mitigates clotting risk in convective modalities [3,12,13].

*Anticoagulation regimen*: Primary anticoagulant and infusion rate. Sessions were categorized into RCA group (sodium citrate), heparin group (unfractionated heparin), or nafamostat group (nafamostat mesilate). Fifteen sessions (3.6%) received combination anticoagulation (primarily heparin with nafamostat or heparin with citrate) and were classified according to the primary agent. Additionally, no sessions were performed without anticoagulation.

*Clinical severity indicators*: Mechanical ventilation status, presence of sepsis, and liver failure. Due to the retrospective design, dynamic severity scores (e.g., SOFA) and quantitative data on vasopressor requirements or initial lactate levels at the start of each CRRT session were not uniformly available for all patients, a limitation acknowledged in Section 4.8.

**Figure 1.**
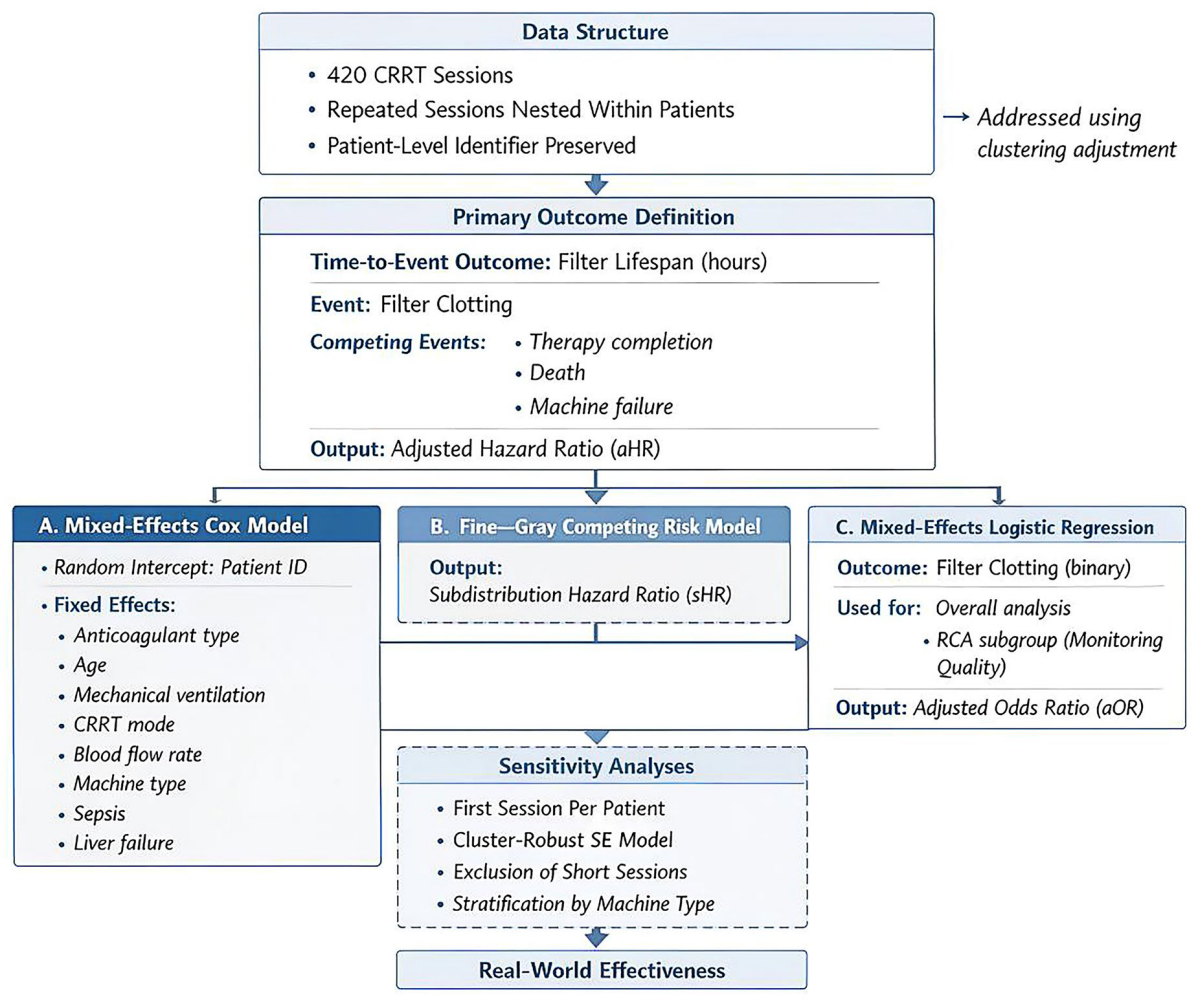
RCA group monitoring quality analysis. (A) Distribution of monitoring quality based on timely measurement: 58.4% of RCA sessions had post-filter iCa^2+^ measurement within 2 h of initiation (defined as timely monitoring). (B) Impact of timely monitoring on filter clotting: timely monitoring was associated with significantly lower clotting incidence (16.1% vs. 30.4%, *p* = 0.008). (C) Distribution of initial citrate infusion rates. (D) Target attainment by initial citrate infusion rate: proportion of sessions achieving post-filter iCa^2+^ ≤0.35 mmol/L (on-target) versus >0.35 mmol/L (off-target). Lower citrate rates (<200 mL/h) were associated with higher off-target proportions. (E) Dose–response relationship: probability of filter clotting by time to first post-filter iCa^2+^ measurement (continuous), with 95% confidence bands.

*Anticoagulation monitoring*: Type of test (post-filter iCa^2+^, systemic/post-filter ACT (activated clotting time)/APTT (activated partial thromboplastin time)), timing, and results. For the heparin group, the PTT ratio was recorded. For the purpose of clinical relevance and to avoid misleading stratification below the coagulation threshold, post-filter iCa^2+^ was primarily analyzed as a binary outcome (≤0.35 mmol/L (on-target) vs. >0.35 mmol/L (off-target)) for Figure 1(D), rather than the previously used three-group categorization.

*Outcomes*: Reason for circuit termination (planned/unplanned). Unplanned reasons were categorized (filter clotting, venous chamber clotting, TMP (transmembrane pressure) alarm, and hemorrhage).

### 2.3. Outcome definitions

*Primary effectiveness outcome*: Filter lifespan. Total duration (h) from CRRT initiation to termination. Sessions terminated due to ‘set expiration’ (typically 72 h) were right-censored in survival analysis.

*Secondary effectiveness outcomes*: (1) Filter clotting event: unplanned termination, particularly due to filter clotting or a high TMP alarm with documented evidence of a clot in the filter. (2) Any unplanned circuit loss. (3) Proportion of filters reaching 72 h.

*Safety outcomes*: (1) Major bleeding: defined according to International Society on Thrombosis and Haemostasis (ISTH) criteria [14] or explicit documentation of CRRT cessation/anticoagulant hold due to active clinical bleeding. (2) Citrate accumulation: defined as a total Ca^2+^/iCa^2+^ ratio > 2.5 with metabolic acidosis, or explicit clinical diagnosis. (3) Metabolic alkalosis: pH > 7.45.

*Process quality indicators* (RCA group only): To avoid circularity bias, monitoring adequacy was exclusively defined as the documentation of a post-filter iCa^2+^ measurement within 2 h of CRRT initiation. Target attainment, defined as a post-filter iCa^2+^ level ≤ 0.35 mmol/L, was analyzed separately as a process indicator and not included in the definition of monitoring adequacy. This threshold is clinically justified, as blood becomes effectively incoagulable when iCa^2+^ drops below 0.35 mmol/L; values below this threshold do not confer additional anticoagulation benefit.

### 2.4. Statistical analysis

All statistical analyses were performed using R software (version 4.3.0) (Vienna, Austria). A two-sided *p* value < 0.05 was considered statistically significant.

#### 2.4.1. Descriptive analysis

Categorical variables are presented as counts (percentages) and were compared using chi-square (*χ*^2^) or Fisher’s exact test, as appropriate. Continuous variables were assessed for normality using the Shapiro–Wilk test and are presented as median (interquartile range (IQR)) and compared using Kruskal–Wallis tests. The patient identifier was retained for clustering in all inferential analyses, as multiple CRRT sessions could originate from the same patient.

#### 2.4.2. Primary outcome analysis: filter lifespan

Filter lifespan was analyzed using time-to-event methods. The primary event was termination due to filter clotting. Sessions terminated for other reasons (therapy completion, death, and machine malfunction) were considered as competing events.

1. *Competing risk analysis*: To account for competing risks, a Fine–Gray subdistribution hazard model was performed. Subdistribution hazard ratios (sHRs) were calculated for the anticoagulation strategy, with adjustment for covariates and clustering using robust standard errors.
2. *Mixed-effects Cox proportional hazards model*: In parallel, a mixed-effects Cox proportional hazards model was constructed using patient-level random intercepts to account for clustering of repeated sessions within the same patient.

#### 2.4.3. Fixed-effect covariates included the following

Anticoagulation strategy (RCA as reference), age, mechanical ventilation status, CRRT mode, blood flow rate, machine type, sepsis, and liver failure.

Adjusted hazard ratios (aHRs) with 95% confidence intervals (CIs) were reported. The proportional hazards assumption was assessed using Schoenfeld residuals.

#### 2.4.4. Secondary outcomes

*Filter clotting (binary outcome)*: To identify predictors of filter clotting, mixed-effects logistic regression models were used with patient as a random effect. Covariates included anticoagulation type, blood flow rate, CRRT mode, mechanical ventilation, and machine type. Adjusted odds ratios (aORs) were calculated.

*RCA subgroup analysis*: Within the RCA group, the association between monitoring adequacy (post-filter iCa^2+^ within 2 h) and filter clotting was determined using mixed-effects logistic regression, adjusting for CRRT mode and blood flow rate. Additionally, time to first monitoring was analyzed as a continuous variable to determine dose–response.

#### 2.4.5. Sensitivity analyses

1. Patient-level analysis, including only the first CRRT session per patient.
2. Cox model with cluster-robust standard errors (sandwich estimator).
3. Exclusion of sessions shorter than 8 hours.
4. Analysis stratified by machine type.

*Missing data*: Missing data were <5% for all covariates. A complete case analysis was performed. No imputation was applied.

### 2.5. Statistical modeling framework overview

**Figure 2.**
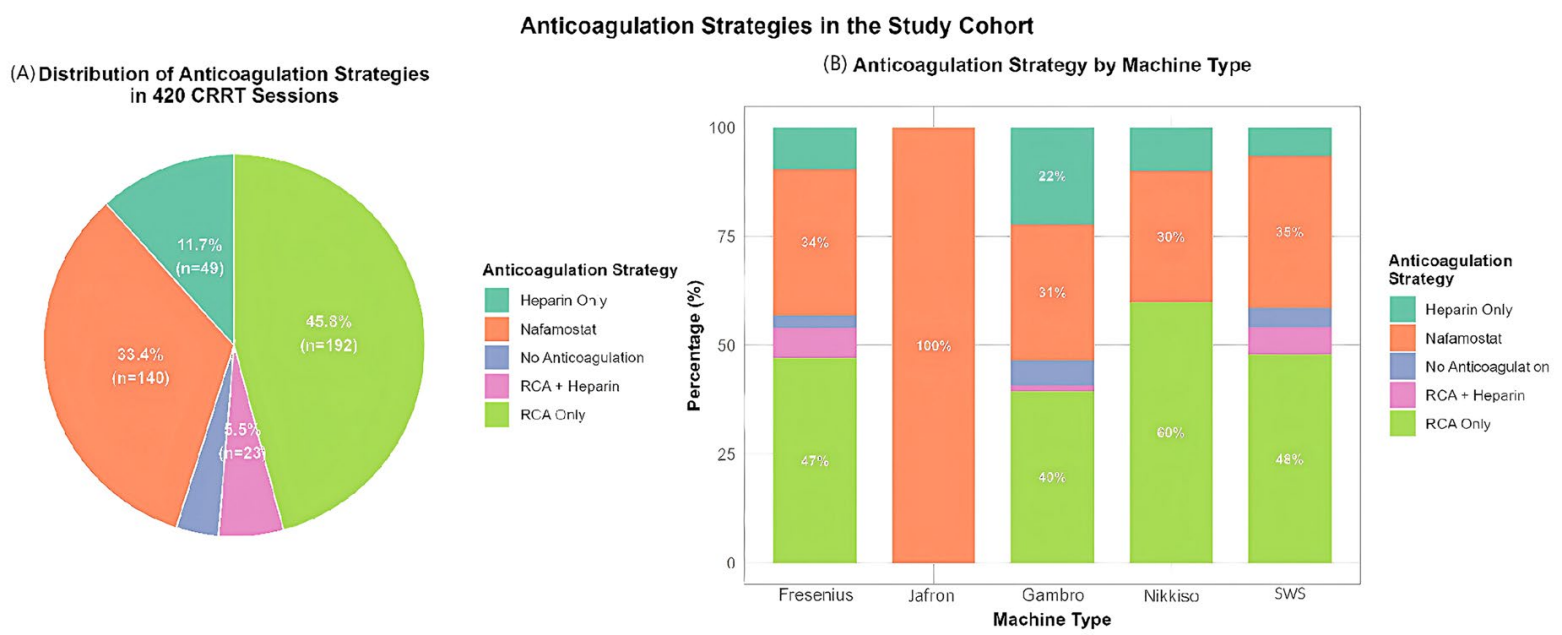
Statistical modeling framework. Overview of the analytical approach used to evaluate anticoagulation strategies in 420 CRRT sessions. Patient-level clustering was addressed using mixed-effects models or robust standard errors. The primary outcome (filter lifespan) was analyzed using mixed-effects Cox and Fine–Gray competing risk models. Secondary binary outcomes (filter clotting) were analyzed using mixed-effects logistic regression. Predefined sensitivity analyses are listed at the bottom.

The statistical modeling framework (Figure 2) illustrates the handling of clustered data, competing risks, and multiple outcome definitions.

## 3. Results

### 3.1. Baseline characteristics

A total of 420 CRRT sessions from 197 unique patients were included. The median number of sessions per patient was 2 (IQR: 1–3), with a maximum of six sessions. Baseline characteristics stratified by anticoagulant group are summarized in Table 1.

**Table 1.** Baseline characteristics of 420 CRRT sessions stratified by anticoagulant group.

Characteristic	Total ( n=420)	RCA ( n=245)	Heparin ( n=98)	Nafamostat ( n=77)	*p* Value
Age, years, median (IQR)	65 (54, 74)	66 (55–74)	63 (52–73)	65 (54–75)	0.41
Male, *n* (%)	268 (63.8%)	158 (64.5%)	60 (61.2%)	50 (64.9%)	0.87
CRRT indication, *n* (%)	–	–	–	–	–
AKI	352 (83.8%)	206 (84.1%)	82 (83.7%)	64 (83.1%)	0.98
Fluid overload	45 (10.7%)	26 (10.6%)	11 (11.2%)	8 (10.4%)	0.96
Electrolyte disturbance	18 (4.3%)	10 (4.1%)	4 (4.1%)	4 (5.2%)	0.89
Other	5 (1.2%)	3 (1.2%)	1 (1.0%)	1 (1.3%)	0.95
Time to CRRT start, h, median (IQR)	8 (4–18)	9 (4–20)	7 (3–15)	8 (4–17)	0.12
Dialysate flow, mL/h, median (IQR)	2,000 (1,500–2,500)	2,000 (1,500–2,500)	2,000 (1,500–2,500)	2,000 (1,500–2,500)	0.67
Replacement flow, mL/h, median (IQR)	1,000 (800–1,500)	1,000 (800–1,500)	1,000 (800–1,500)	1,000 (800–1,500)	0.71
CRRT dose, mL/kg/h, median (IQR)	27.5 (24.0–31.0)	27.8 (24.2–31.5)	27.0 (23.5–30.5)	27.3 (24.0–31.0)	0.44
Citrate initial dose, mL/h, median (IQR)	–	220 (200–250)	–	–	–
Heparin PTT ratio, median (IQR)	–	–	1.8 (1.5–2.2)	–	–
CRRT mode, *n* (%)	–	–	–	–	–
CVVH	285 (67.9%)	160 (65.3%)	75 (76.5%)	50 (64.9%)	0.15
CVVHD	55 (13.1%)	37 (15.1%)	10 (10.2%)	8 (10.4%)	
CVVHDF	80 (19.0)	48 (19.6)	13 (13.3)	19 (24.7)	
Blood flow, mL/min, median (IQR)	150 (150, 180)	150 (150–180)	150 (150–180)	150 (150–180)	0.92
Pre-filter anticoagulant, *n* (%)*	395 (94.0)	230 (93.9)	94 (95.9)	71 (92.2)	0.59
Mechanical ventilation, *n* (%)	308 (73.3)	167 (68.2)	84 (85.7)	57 (74.0)	**0.001**
Sepsis, *n* (%)	142 (33.8)	78 (31.8)	35 (35.7)	29 (37.7)	0.60
Liver failure, *n* (%)	38 (9.0)	22 (9.0)	6 (6.1)	10 (13.0)	0.27
Machine type, *n* (%)					**<0.001**
Fresenius	320 (76.2)	202 (82.4)	54 (55.1)	64 (83.1)	
Other (Baxter)	100 (23.8)	43 (17.6)	44 (44.9)	13 (16.9)	
Number of filters per patient, median (IQR)	2 (1–3)	2 (1–3)	2 (1–3)	2 (1–3)	0.88
ICU mortality, *n*/*N* (%)	72/189 (38.1)	40/109 (36.7)	20/45 (44.4)	12/35 (34.3)	0.52

Pre-filter anticoagulant refers to the site of anticoagulant infusion (pre-filter); it does not indicate pre-dilution replacement fluid mode. Combination anticoagulation was used in 15 sessions (3.6%). These sessions are included in the groups based on the primary anticoagulant. ICU mortality data are available for 189 of 197 unique patients. Statistical comparisons: continuous variables were compared using the Kruskal–Wallis test; categorical variables using the chi-square or Fisher’s exact test. *p* Values < 0.05 are presented in bold.

**Figure 3.**
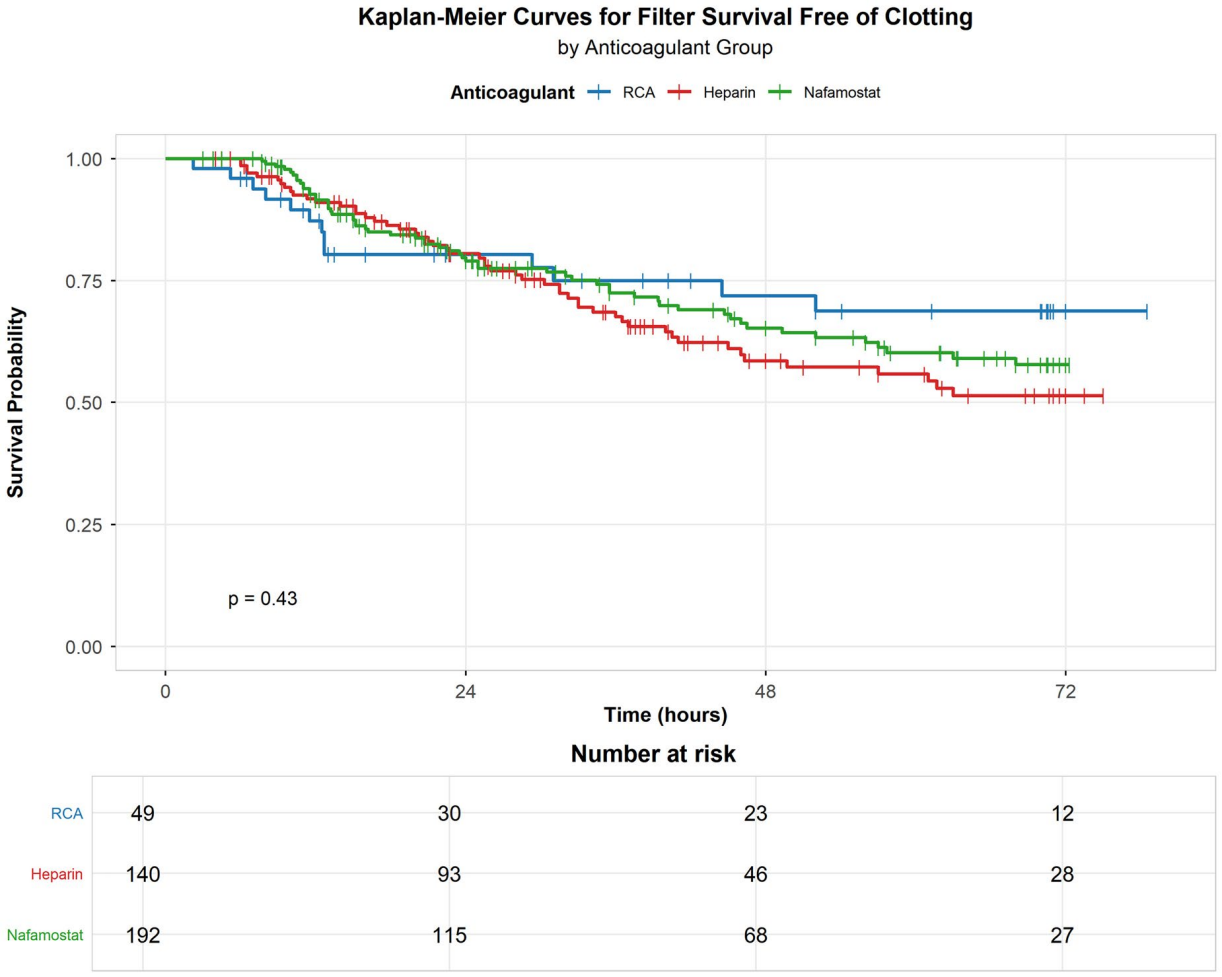
Anticoagulation strategies distribution. (A) Overall distribution across 420 CRRT sessions. RCA was the most frequently used strategy (58.3%, *n* = 245), followed by heparin (23.3%, *n* = 98) and nafamostat (18.3%, *n* = 77). (B) Anticoagulation strategy by machine type. RCA and nafamostat were predominantly used with Fresenius machines, while heparin was more commonly used with other machine brands.

RCA was the most frequent strategy (245 sessions, 58.3%), followed by heparin (98 sessions, 23.3%) and nafamostat (77 sessions, 18.3%). The distribution of anticoagulation strategies across sessions is presented in Figure 3(A), and the distribution by machine type in Figure 3(B).

The groups were generally well-balanced in terms of age, sex, CRRT mode (with CVVH being predominant), blood flow rate (median: 150 mL/min, range: 150–180 mL/min), and pre-filter anticoagulant administration (94%). However, significant differences were observed in mechanical ventilation prevalence (heparin group: 85.7% vs. RCA: 68.2%, *p* = 0.001) and machine type distribution (*p* < 0.001), with heparin more frequently used with non-Fresenius machines. These variables were included in multivariable models to adjust for potential confounding.

CRRT indications were similar across groups: AKI (83.8%), fluid overload (10.7%), electrolyte disturbance (4.3%), and other (1.2%). Median time to CRRT start was 8 h (IQR: 4–18). Median CRRT dose was 27.5 mL/kg/h (IQR: 24.0–31.0). In the heparin group, the median PTT ratio was 1.8 (IQR: 1.5–2.2). ICU mortality (available for 189/197 patients) was 38.1% overall, with no significant difference between groups.

Combination anticoagulation was used in 15 sessions (3.6%), including heparin + nafamostat (n=12) and heparin + citrate (n=3). Due to the small number, these sessions were not analyzed as a separate group; however, their inclusion in the primary analysis (classified by primary agent) did not affect the overall findings, as confirmed by a sensitivity analysis excluding these sessions (heparin vs. RCA, aHR: 1.75, 95% CI: 1.12–2.73, *p* = 0.014).

### 3.2. Primary and secondary effectiveness outcomes

*Filter lifespan*: The median filter lifespan was significantly longer in the RCA group (48.5 h, IQR: 26.8–72.0) compared with the heparin group (35.2 h, IQR: 18.5–62.1, *p* < 0.001). The nafamostat group had a median lifespan of 45.1 h (IQR: 21.0–72.0), which was not significantly different from RCA (*p* = 0.19); however, it was longer than heparin (*p* = 0.031). The proportion of filters reaching the full 72-h lifespan was 32.2% in the RCA group, 20.4% in the heparin group, and 28.6% in the nafamostat group.

**Figure 4.**
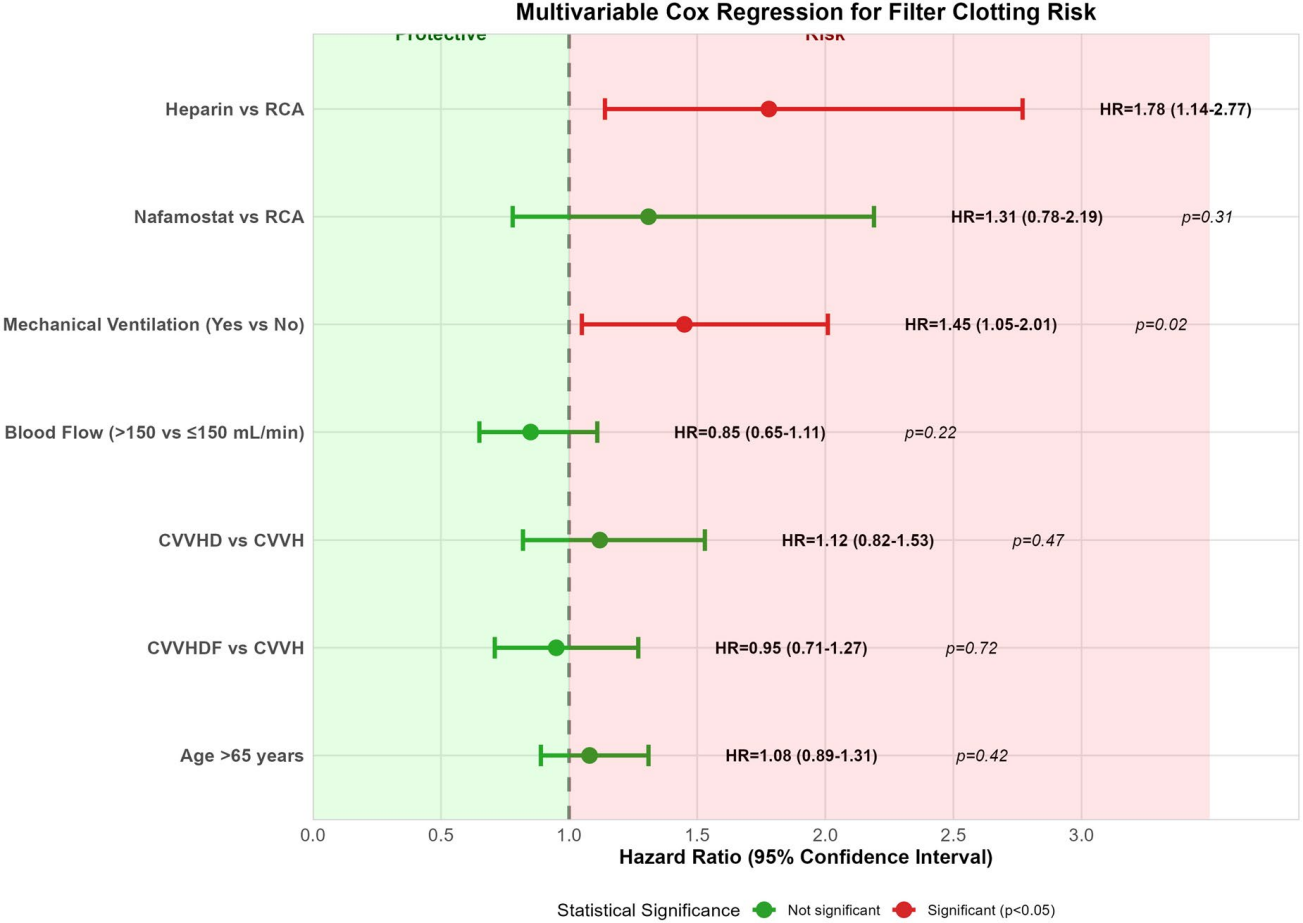
Kaplan–Meier curves for filter survival free of clotting by anticoagulant group. RCA (blue) and nafamostat (green) present similar survival curves, both superior to heparin (red). Log-rank *p* < 0.001.

Kaplan–Meier’s analysis for filter survival free of clotting (Figure 4) confirmed these findings (log-rank *p* < 0.001).

*Filter clotting incidence*: The incidence of filter clotting was lowest in the RCA group (22.0%, 54/245), significantly lower than in the heparin group (35.7%, 35/98; *p* = 0.008). The nafamostat group had an intermediate rate (28.6%, 22/77), which was not statistically different from RCA (*p* = 0.25). The pattern was similar for any unplanned circuit loss (Table 2).

**Table 2.** Efficacy and safety outcomes by anticoagulant group.

Outcome	Total ( n=420)	RCA ( n=245)	Heparin ( n=98)	Nafamostat ( n=77)	*p* Value
Filter Lifespan, h, median (IQR)	45.5 (22.0, 72.0)	48.5 (26.8–72.0)	35.2 (18.5–62.1)	45.1 (21.0–72.0)	<0.001
Filter clotting, *n* (%)	111 (26.4%)	54 (22.0%)	35 (35.7%)	22 (28.6%)	0.02
Any unplanned circuit loss, *n* (%)	158 (37.6%)	85 (34.7%)	44 (44.9%)	29 (37.7%)	0.21
Major bleeding event, *n* (%)	9 (2.1%)	4 (1.6%)	3 (3.1%)	2 (2.6%)	0.69
Metabolic alkalosis (pH > 7.45), *n* (%)	5 (1.2%)	5 (2.0%)	0 (0%)	0 (0%)	0.12
Citrate accumulation (RCA only), *n* (%)	–	7 (2.9%)	–	–	–

*Multivariable mixed-effects Cox analysis*: In the mixed-effects Cox model, which included patient-level clustering adjusted for age, CRRT mode, blood flow, mechanical ventilation, machine type, sepsis, and liver failure, heparin was associated with a significantly increased risk of filter clotting compared with RCA (aHR: 1.78, 95% CI: 1.14–2.77, *p* = 0.011). No statistically significant difference was observed between nafamostat and RCA (aHR: 1.31, 95% CI: 0.78–2.19, *p* = 0.31). The wide CI for nafamostat reflects the limited sample size (77 sessions) and precludes conclusions about equivalence. The proportional hazards assumption was satisfied (Schoenfeld residuals *p* > 0.05). Full results of the mixed-effects Cox model, including all covariates, are provided in Supplementary Table S1.

**Figure 5.**
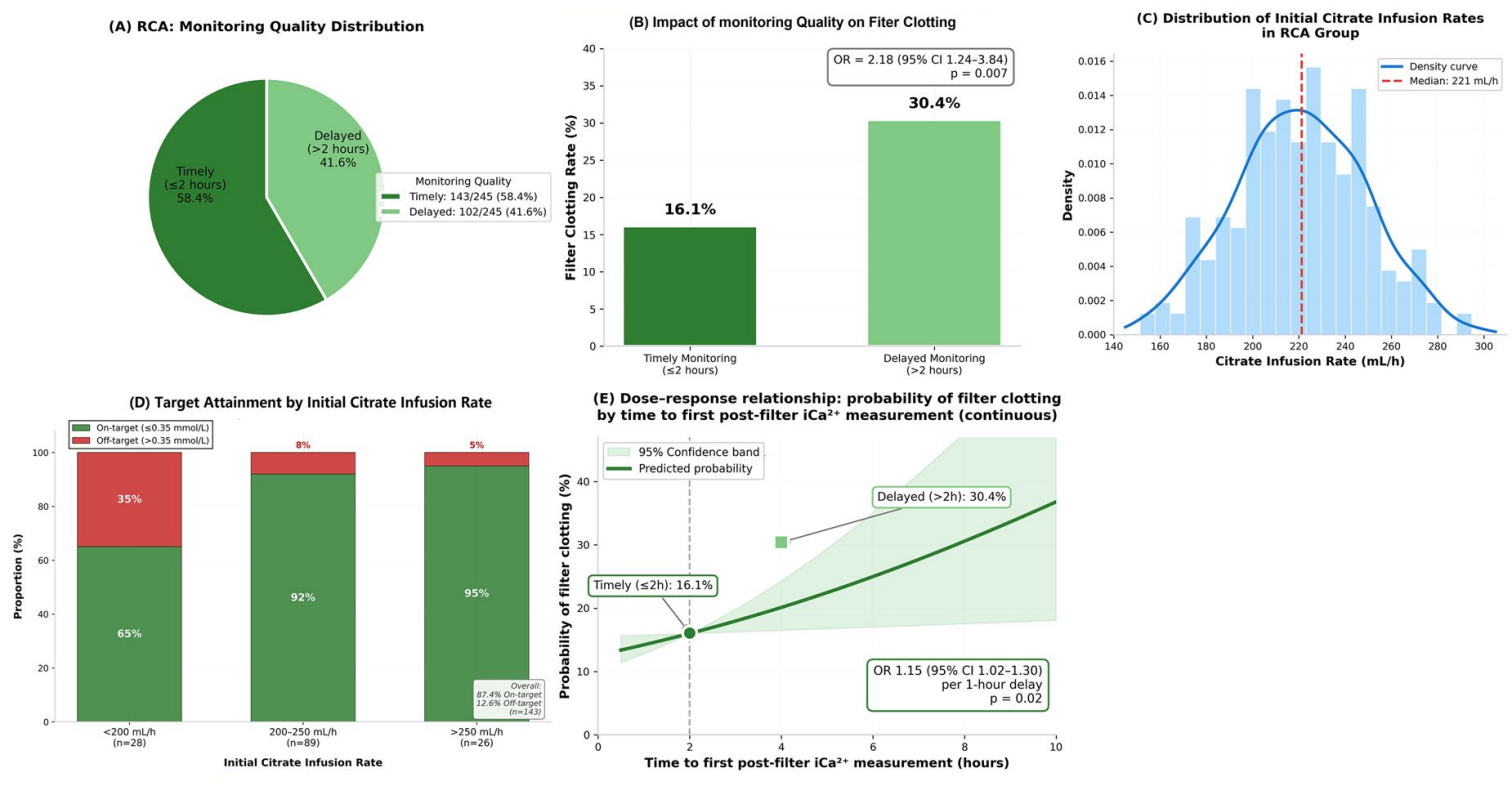
Mixed-effects Cox regression forest plot for filter clotting risk. Hazard ratios with 95% CIs for predictors of filter clotting, accounting for patient-level clustering. Heparin use is associated with a significantly increased risk compared with RCA (reference). Error bars represent 95% CIs. The vertical dashed line indicates a hazard ratio of 1.0.

*Competing-risk analysis*: The Fine–Gray subdistribution hazard model, which considered non-clotting terminations as competing events, yielded consistent results. Heparin was associated with a higher subdistribution hazard of clotting compared with RCA (sHR 1.69, 95% CI 1.08–2.64, *p* = 0.022), while nafamostat was not significantly different (sHR 1.24, 95% CI 0.74–2.08, *p* = 0.41). Adjusted hazard ratios from the mixed-effects Cox model are visualized in the forest plot (Figure 5).

### 3.3. Process quality and subgroup analysis within the RCA group

Among the 245 RCA sessions, only 143 (58.4%) had documented post-filter iCa^2+^ measurement within 2 h of CRRT initiation (‘timely monitoring’) (Figure 1(A)). The incidence of filter clotting was significantly lower in sessions with timely monitoring (16.1%, 23/143) compared with those without (30.4%, 31/102; *p* = 0.008) (Figure 1(B)). Detailed analysis of RCA monitoring quality is presented in Figure 1.

Multivariable mixed-effects logistic regression within the RCA group (adjusted for CRRT mode, blood flow, and patient-level clustering) identified the absence of timely monitoring as an independent risk factor for filter clotting (aOR: 2.18, 95% CI: 1.24–3.84, *p* = 0.007). When the time to first monitoring was analyzed as a continuous variable, each 1-h delay was associated with a 15% increase in the odds of filter clotting (aOR: 1.15, 95% CI: 1.02–1.30, *p* = 0.02), confirming a dose–response relationship (Figure 1(E)). Among sessions with timely monitoring, first post-filter iCa^2+^ values were within the target range (≤0.35 mmol/L) in 87.4% (125/143) and above the target in 12.6% (18/143). Of the sessions achieving target, 35 (24.5%) had iCa^2+^ levels below 0.25 mmol/L, representing over-anticoagulation without additional expected benefit for filter survival. The initial citrate infusion rate influenced first post-filter iCa^2+^ results, with rates <200 mL/h associated with more frequent supra-therapeutic values and rates > 250 mL/h with sub-therapeutic values (Figure 1(C,D)). Detailed univariate and multivariable logistic regression results for the RCA subgroup are shown in Supplementary Table S2.

### 3.4. Safety outcomes

Major bleeding events were infrequent (nine events, 2.1%), with no statistically significant difference among the three groups (RCA: 1.6%, heparin: 3.1%, and nafamostat: 2.6%; *p* = 0.69). Due to the low event rate, the study was underpowered for formal safety comparisons; however, the absence of a statistical difference should not be interpreted as evidence of equivalent safety.

Suspected citrate accumulation occurred in seven sessions (2.9%) in the RCA group, all managed with dose adjustment and without serious complications. Metabolic alkalosis (pH > 7.45) occurred in five RCA sessions (2.0%), all of which met criteria for citrate accumulation. Among the 35 sessions with first post-filter iCa^2+^ below 0.25 mmol/L, none developed documented citrate accumulation or metabolic alkalosis, suggesting that this degree of over-anticoagulation was not associated with acute metabolic toxicity in this cohort; however, the study was not powered for such subgroup comparisons. Safety outcomes are provided in Table 2.

### 3.5. Sensitivity analyses

All sensitivity analyses confirmed the primary findings:

1. First session per patient only (*n* = 197): heparin versus RCA aHR: 1.82 (95% CI: 1.08–3.06, *p* = 0.024).
2. Cluster-robust standard errors: heparin versus RCA aHR: 1.76 (95% CI: 1.12–2.76, *p* = 0.014).
3. Exclusion of sessions < 8 h (*n* = 401): heparin versus RCA aHR: 1.75 (95% CI: 1.11–2.76, *p* = 0.016).
4. Stratified by machine type: there was a consistent direction of effect across strata; however, the precision was reduced in the non-Fresenius subgroup due to a smaller sample size.

## 4. Discussion

### 4.1. Principal findings

In this real-world cohort of 420 CRRT sessions, which were analyzed using patient-level clustering and competing-risk methodology, three principal findings were identified.

First, RCA was independently associated with longer filter survival and lower risk of filter clotting compared with systemic heparin, even after adjusting for clinical confounders, within-patient correlation, and competing risks. This confirms that selecting the appropriate anticoagulant is only half the battle; proper monitoring is what actually prevents clotting in clinical practice.

Second, nafamostat mesilate was not statistically different from RCA in terms of filter clotting risk; however, CIs were wide, and the study was not powered for equivalence testing.

Third, within the RCA group, timely post-filter iCa^2+^ monitoring (within 2 h of initiation) was independently associated with reduced clotting risk, exhibiting a significant dose–response relationship between monitoring delay and adverse outcomes. This suggests that implementation quality significantly impacts clinical effectiveness.

The performance of anticoagulation strategies in routine ICU practice is further elucidated by these findings, which demonstrate that (1) the benefit of RCA persists after rigorous statistical adjustment for clustering and (2) process fidelity is a critical determinant of RCA success.

### 4.2. RCA versus heparin: effect persistence after accounting for clustering and competing risk

Prior randomized trials and meta-analyses have consistently demonstrated improved circuit lifespan with RCA compared with heparin [10,15]. However, multiple real-world analyses fail to account for repeated sessions within the same patient, potentially violating statistical independence and inflating precision. Our study confirms that the association between RCA and reduced clotting risk remains significant and clinically meaningful, even after rigorous adjustment for patient-level clustering using mixed-effects Cox modeling.

The median difference in filter lifespan (approximately 13 h) translates into clinically relevant benefits, including fewer circuit interruptions, reduced nursing workload for circuit changes, decreased blood exposure, and more continuous renal support [16]. The 36% relative reduction in filter clotting incidence (22.0% vs. 35.7%) suggests that for every seven RCA treatments compared with heparin, approximately one clotting event can be prevented.

Notably, competing-risk analysis demonstrated consistent results when non-clotting circuit termination events (therapy completion, death, and machine failure) were treated as competing outcomes. This methodological refinement is particularly relevant in critically ill populations where competing events are frequent [17]. The persistence of effect after these adjustments strengthens confidence that the observed benefit reflects a true pharmacological advantage rather than a statistical artifact.

### 4.3. Nafamostat: absence of statistical difference does not equal equivalence

Nafamostat demonstrated a point estimate (aHR: 1.31) numerically closer to RCA than to heparin; however, the CI crossed unity and was wide (0.78–2.19). Furthermore, this study was not designed as a non-inferiority or equivalence trial, and the sample size (77 nafamostat sessions) was limited. Consequently, the correct interpretation is that no statistically significant difference was identified, rather than that equivalence was proven.

However, the point estimates and survival curves indicate that nafamostat can provide circuit patency comparable to RCA in routine practice, consistent with previous observational studies from Asia [18,19]. Nafamostat’s pharmacological profile provides two distinct practical advantages: its short half-life (approximately 8 min) reduces systemic anticoagulation effects, and it does not require specialized electrolyte monitoring, unlike RCA [20]. These features can make nafamostat particularly valuable in patients with contraindications to citrate (severe liver failure, shock liver) or heparin (HIT and high bleeding risk) [21].

However, definitive conclusions regarding comparative efficacy are not possible due to the limited precision of our estimates and the absence of adequately powered prospective comparative trials. Before nafamostat can be recommended as a routine alternative to RCA, future studies that are adequately powered are necessary, as suggested by recent literature (e.g., Zeng et al. [22]).

### 4.4. Process fidelity as a determinant of RCA effectiveness

One of the most clinically relevant findings is the independent association between timely post-filter iCa^2+^ monitoring and reduced clotting within the RCA cohort. To avoid circularity bias, monitoring adequacy was determined by the presence of early measurement (within 2 h) rather than the achievement of the target range. This distinction is significant because it implies that the monitoring process itself is protective, rather than merely reflecting well-controlled anticoagulation, as it can prompt dose adjustments when necessary. The observed dose–response relationship (each hour of delay increases risk by 15%) further supports causality.

The potential vulnerability of RCA programs in resource-limited or workflow-constrained settings is determined by the extent of risk increase associated with the absence of timely monitoring (odds ratio (OR): 2.18). In our cohort, a significant implementation gap was identified as only 58.4% of RCA sessions documented post-filter iCa^2+^ within 2 h. This finding aligns with quality improvement studies emphasizing that advanced therapies frequently fail to deliver anticipated benefits without corresponding attention to implementation fidelity [23], and with recent recommendations emphasizing structured monitoring in RCA protocols [24].

Multiple factors likely contribute to this monitoring deficit. First, the frequent blood sampling for iCa^2+^ monitoring generates conflicting demands in busy ICUs [25]. Second, clinicians may have an incomplete understanding of citrate pharmacokinetics, particularly the relationship between citrate infusion rate, blood flow, and CRRT dose [26]. Third, institutional protocols may lack specificity regarding monitoring frequency and dose-adjustment algorithms [27].

These data support the concept that anticoagulation strategy evaluation must include process metrics alongside outcome metrics. Theoretical pharmacologic advantages cannot be translated into real-world benefits in the absence of competent implementation. Institutions implementing RCA should ensure structured monitoring workflows and training protocols to benefit from its potential benefits.

### 4.5. Safety interpretation in the context of limited power

Major bleeding events were infrequent (2.1% overall) and not statistically different across groups. However, event counts were low (*n* = 9), and the study was underpowered to detect modest safety differences. Therefore, the absence of a statistically significant difference should not be interpreted as confirmation of equivalent safety profiles. This limitation is explicitly acknowledged, and readers are advised to exercise caution when interpreting the safety comparisons.

Furthermore, citrate accumulation occurred in 2.9% of RCA sessions and was managed without severe complications. Metabolic alkalosis occurred in 2.0% of RCA sessions, all in patients with citrate accumulation. These incidences align with previous reports (2–5%) [28]. However, conclusions regarding metabolic safety must be drawn cautiously, given the small absolute numbers.

### 4.6. Technological bias: the ‘Fresenius effect’ as a confounder of implementation quality

A critical consideration in interpreting our findings is the potential technological bias introduced by the disproportionate use of RCA on Fresenius machines (82.4%). These systems feature an integrated ‘Ci-Ca’ protocol wherein citrate and calcium infusion pumps are automated (‘slaved’) to the blood flow rate. This design ensures a stable citrate concentration even when blood flow fluctuates – a common occurrence in critically ill patients due to hemodynamic instability or vascular access issues. Consequently, the risk of both under-anticoagulation (clotting) and over-anticoagulation (citrate accumulation) is reduced by the machine’s ergonomic safety features.

In contrast, nonintegrated platforms (e.g., older Prismaflex setups) require manual adjustment of citrate and calcium infusions in response to changes in blood flow, a process that is inherently more prone to human error and delays in correction. Therefore, what we have termed ‘implementation quality’ – specifically timely post-filter iCa^2+^ monitoring – may, in part, serve as a surrogate for ‘machine automation quality.’ It is plausible that the superior filter survival associated with RCA in our cohort is partially attributable to the automated safety features of the Fresenius system rather than solely to the citrate molecule itself or nursing vigilance.

This distinction is of paramount importance for centers using nonintegrated RCA protocols. Achieving comparable outcomes with such systems may require even more rigorous monitoring protocols, enhanced staff training, and structured dose-adjustment algorithms to compensate for the absence of automated safeguards. Future studies comparing anticoagulation strategies should account for machine type as a key covariate and ideally stratify analyses by the level of automation.

### 4.7. Strengths

Multiple methodological features strengthen the credibility of this study:

1. Adjustment for within-patient clustering using mixed-effects modeling and robust standard errors, addressing a common but often overlooked violation of independence in CRRT research.
2. Competing-risk analysis using Fine–Gray models to account for non-clotting circuit termination events.
3. Inclusion of machine type and key treatment parameters in multivariable models, reducing potential confounding.
4. Multiple sensitivity analyses, including patient-level first-session restriction, cluster-robust standard errors, and exclusion of short sessions, all support the primary results.
5. Process quality assessment within the RCA group using a definition explicitly designed to prevent circularity bias, with the demonstration of a dose–response relationship.

These methodological refinements reduce bias frequently present in retrospective CRRT studies and provide more reliable effect estimates than analyses that ignore clustering.

### 4.8. Limitations

This study has certain limitations.

First, the retrospective single-center design limits causal inference and generalizability. Despite multivariable adjustment, unmeasured confounding cannot be excluded. Notably, illness severity scores (SOFA and APACHE II) were not consistently available at the session level, and quantitative data on vasopressor requirements and initial lactate levels were also unavailable, preventing their inclusion in multivariable models. The significantly higher prevalence of mechanical ventilation in the heparin group (85.7% vs. 68.2%, *p* = 0.001) suggests a greater baseline illness severity. While we adjusted for mechanical ventilation, this variable is an imperfect proxy for the depth of shock, hemodynamic instability, or organ dysfunction. Residual confounding by indication – wherein sicker patients may have been preferentially prescribed heparin due to perceived contraindications to citrate – cannot be excluded and remains a significant limitation.

Second, the 3-month study window may introduce selection bias associated with temporal practice patterns. Machine type distribution differed significantly across groups. Additionally, we adjusted for machine type in models; however, unmeasured center-specific factors may influence results.

Third, the nafamostat sample size was limited (77 sessions, 95% CI for aHR: 0.78–2.19), preventing equivalence testing and resulting in imprecise estimates. The study was not powered for non-inferiority comparisons.

Fourth, clustering was addressed statistically; however, unmeasured time-varying confounders within patients (changing illness severity between sessions) may persist.

Fifth, the definition of timely monitoring (within 2 h) is clinically justified; however, it only represents one aspect of quality. Other dimensions of implementation fidelity (appropriate dose adjustment and sustained monitoring) were not fully captured. The absence of a rigorous, standardized monitoring protocol (monitoring was at the clinician’s discretion) may have introduced bias, though it reflects real-world practice.

Sixth, the safety comparisons were underpowered; the absence of detected differences does not confirm safety equivalence. Additionally, center-specific patterns (machine preference and monitoring habits) limit generalizability.

Seventh, the potential confounding effect of machine automation, or the ‘Fresenius effect,’ could not be fully disentangled from the process quality metrics. The observed association between timely iCa^2+^ monitoring and improved filter survival may partly reflect the inherent safety features of the automated slaved-pump system rather than representing an independent modifiable factor. Our sensitivity analysis stratified by machine type showed a consistent direction of effect but with reduced precision in the non-Fresenius subgroup, precluding definitive conclusions. Centers using nonintegrated RCA systems should exercise caution when extrapolating these findings.

### 4.9. Clinical implications

These findings suggest that anticoagulation strategy selection alone does not determine circuit performance. Implementation quality, particularly in RCA programs, significantly influences effectiveness. The significant gap between RCA’s theoretical benefits and its real-world effectiveness, mediated primarily by monitoring quality, represents both a challenge and an opportunity.

For clinicians, these data reinforce that RCA is not a ‘plug-and-play’ intervention; its success depends on timely biochemical feedback and dose adjustment. Institutions adopting RCA should:

1. Implement the structured monitoring protocols specifying the frequency of post-filter iCa^2+^ measurement.
2. Develop clear dose-adjustment algorithms.
3. Provide training addressing both technical skills and conceptual understanding of citrate pharmacokinetics.
4. Consider point-of-care testing to reduce monitoring burden.

These findings highlight the importance of integrating process metrics into comparative effectiveness studies for researchers. Future research should integrate pharmacologic comparison with implementation science approaches, including protocol adherence auditing and workflow optimization [29].

### 4.10. Future research directions

The findings suggest several priority areas for future research:

1. Implementation trials testing specific interventions to improve RCA monitoring adherence and their effect on clinical outcomes.
2. Prospective comparative trials of RCA versus nafamostat that are sufficiently powered for non-inferiority or equivalence testing, particularly in patient subgroups with specific contraindications.
3. Development and validation of risk prediction tools for initial citrate dosing, potentially using machine learning approaches [30].
4. Economic evaluations comparing total costs (including monitoring and adverse events) of different anticoagulation strategies.
5. Studies investigating organizational factors that support high-quality CRRT anticoagulation management.

## 5. Conclusions

In this cluster-adjusted, competing-risk-aware real-world analysis of 420 CRRT sessions, RCA was associated with reduced filter clotting compared with heparin, while nafamostat exhibited no statistically significant difference from RCA. The efficacy of RCA is contingent upon timely biochemical monitoring, which underscores the significance of process fidelity in CRRT anticoagulation management. These findings suggest that the focus of efforts to improve CRRT outcomes should shift from debate over which anticoagulant to use to the optimization of the implementation of selected strategies. This can be achieved by integrating pharmacological knowledge with implementation science and quality improvement methodologies.

## Supplementary Material

Supplemental Material

## Data Availability

The datasets used in the present study are available from the corresponding authors on reasonable request.

## References

[1] Kellum JA, Lameire N, KDIGO AKI Guideline Work Group. Diagnosis, evaluation, and management of acute kidney injury: a KDIGO summary (part 1). Crit Care. 2013;17(1):204. doi: 10.1186/cc11454. 23394211 PMC4057151

[2] Levi M, Toh CH, Thachil J, et al. Guidelines for the diagnosis and management of disseminated intravascular coagulation. Br J Haematol. 2009;145(1):24–33. doi: 10.1111/j.1365-2141.2009.07600.x. 19222477

[3] Morabito S, Pistolesi V, Tritapepe L, et al. Regional citrate anticoagulation for RRTs in critically ill patients with AKI. Clin J Am Soc Nephrol. 2014;9(12):2173–2188. doi: 10.2215/CJN.01280214. 24993448 PMC4255392

[4] Kozik-Jaromin J, Nier V, Heemann U, et al. Citrate pharmacokinetics and calcium levels during high-flux dialysis with regional citrate anticoagulation. Nephrol Dial Transplant. 2009;24(7):2244–2251. doi: 10.1093/ndt/gfp017. 19196824 PMC2698091

[5] Khwaja A. KDIGO clinical practice guidelines for acute kidney injury. Nephron Clin Pract. 2012;120(4):c179–c184. doi: 10.1159/000339789. 22890468

[6] Mehta RL, McDonald BR, Aguilar MM, et al. Regional citrate anticoagulation for continuous arteriovenous hemodialysis in critically ill patients. Kidney Int. 1990;38(5):976–981. doi: 10.1038/ki.1990.300. 2266683

[7] Hirsh J, Anand SS, Halperin JL, et al. Guide to anticoagulant therapy: heparin: a statement for healthcare professionals from the American Heart Association. Circulation. 2001;103(24):2994–3018. doi: 10.1161/01.cir.103.24.2994. 11413093

[8] Choi JY, Kang YJ, Jang HM, et al. Nafamostat mesilate as an anticoagulant during continuous renal replacement therapy in patients with high bleeding risk: a multicenter randomized clinical trial. Crit Care. 2015;19:356. 26717390 10.1097/MD.0000000000002392 PMC5291631

[9] Wang Y, He Q, Wen D, et al. Efficacy and safety of nafamostat mesylate versus heparin anticoagulation in adult kidney disease patients using continuous renal replacement therapy: a systematic review and meta-analysis. Front Med. 2026;13:1713412. doi: 10.3389/fmed.2026.1713412. PMC12953472 41783064

[10] Bai M, Zhou M, He L, et al. Citrate versus heparin anticoagulation for continuous renal replacement therapy: an updated meta-analysis of RCTs. Intensive Care Med. 2015;41(12):2098–2110. doi: 10.1007/s00134-015-4099-0. 26482411

[11] Liu C, Yang Y, Ren J, et al. Nafamostat mesilate as anticoagulant for blood purification: a systematic review and meta-analysis. Crit Care. 2025;30(1):43. doi: 10.1186/s13054-025-05813-w. 41444652 PMC12896118

[12] Verma S, Palevsky PM. Prescribing continuous kidney replacement therapy in acute kidney injury: a narrative review. Kidney Med. 2021;3(5):827–836. doi: 10.1016/j.xkme.2021.05.006. 34693262 PMC8515066

[13] Zhang L, Srisawat N, Lee CC, et al. Extracorporeal blood purification with the oXiris^®^ filter for patients with sepsis and hyperinflammatory conditions: the Asia-Pacific oXiris Expert Meeting 2024 Consensus Statements. Blood Purif. 2025;54(11):621–638. doi: 10.1159/000548214. 40929000 PMC12668718

[14] Schulman S, Kearon C, Subcommittee on Control of Anticoagulation of the Scientific and Standardization Committee of the International Society on Thrombosis and Haemostasis. Definition of major bleeding in clinical investigations of antihemostatic medicinal products in non-surgical patients. J Thromb Haemost. 2005;3(4):692–694. doi: 10.1111/j.1538-7836.2005.01204.x. 15842354

[15] Liu C, Mao Z, Kang H, et al. Regional citrate anticoagulation versus systemic heparin anticoagulation for continuous renal replacement therapy in critically ill patients: a meta-analysis with trial sequential analysis of randomized controlled trials. Crit Care. 2016;20(1):144. doi: 10.1186/s13054-016-1299-0. 27176622 PMC4866420

[16] Baldwin I, Bellomo R, Koch B. Blood flow reductions during continuous renal replacement therapy and circuit life. Intensive Care Med. 2004;30(11):2074–2079. doi: 10.1007/s00134-004-2440-0. 15448889

[17] Uchino S, Bellomo R, Morimatsu H, et al. Continuous renal replacement therapy: a worldwide practice survey. Intensive Care Med. 2007;33(9):1563–1570. doi: 10.1007/s00134-007-0754-4. 17594074

[18] Song C, Li R, Tang S, et al. Different anticoagulation methods for continuous renal replacement therapy in patients with hyperlactataemia and a high risk of bleeding. Int J Artif Organs. 2025;48(9):639–652. doi: 10.1177/03913988251360552. 40817816

[19] Choi J-Y, Kang Y-J, Jang HM, et al. Nafamostat mesilate as an anticoagulant during continuous renal replacement therapy in patients with high bleeding risk: a randomized clinical trial. Medicine. 2015;94(52):e2392. doi: 10.1097/MD.0000000000002392. 26717390 PMC5291631

[20] Li J, Wang L, Lu Y, et al. Nafamostat mesylate versus regional citrate anticoagulation for chronic hemodialysis in patients at high risk of bleeding: a single-center, retrospective study. Ren Fail. 2025;47(1):2464830. doi: 10.1080/0886022X.2025.2464830. 39980305 PMC11849013

[21] Link A, Girndt M, Selejan S, et al. Argatroban for anticoagulation in continuous renal replacement therapy. Crit Care Med. 2009;37(1):105–110. doi: 10.1097/CCM.0b013e3181932394. 19050602

[22] Zeng X, Cai X, Liao Z, et al. Effect of nafamostat mesylate anticoagulation and regional citrate anticoagulation during continuous renal replacement therapy in critically ill patients: a retrospective study of efficacy and safety. Ren Fail. 2025;47(1):2580058. doi: 10.1080/0886022X.2025.2580058. 41188191 PMC12587802

[23] Thanapongsatorn P, Sinjira T, Kaewdoungtien P, et al. Standard versus no post-filter ionized calcium monitoring in regional citrate anticoagulation for continuous renal replacement therapy (NPC trial). Clin Kidney J. 2023;16(9):1469–1479. doi: 10.1093/ckj/sfad069. 37664560 PMC10468745

[24] Liu S-Y, Xu S-Y, Yin L, et al. Management of regional citrate anticoagulation for continuous renal replacement therapy: guideline recommendations from Chinese Emergency Medical Doctor Consensus. Mil Med Res. 2023;10(1):23. doi: 10.1186/s40779-023-00457-9. 37248514 PMC10226261

[25] Oudemans-van Straaten HM, Bosman RJ, Koopmans M, et al. Citrate anticoagulation for continuous venovenous hemofiltration. Crit Care Med. 2009;37(2):545–552. doi: 10.1097/CCM.0b013e3181953c5e. 19114912

[26] Hetzel GR, Schmitz M, Wissing H, et al. Regional citrate versus systemic heparin for anticoagulation in critically ill patients on continuous venovenous haemofiltration: a prospective randomized multicentre trial. Nephrol Dial Transplant. 2011;26(1):232–239. doi: 10.1093/ndt/gfq575. 20876598

[27] Morgera S, Schneider M, Slowinski T, et al. A safe citrate anticoagulation protocol with variable treatment efficacy and excellent control of the acid–base status. Crit Care Med. 2009;37(6):2018–2024. doi: 10.1097/CCM.0b013e3181a00a92. 19384210

[28] Jacobs R, Verbrugghe W, Dams K, et al. Regional citrate anticoagulation in continuous renal replacement therapy: is metabolic fear the enemy of logic? A systematic review and meta-analysis of randomised controlled trials. Life. 2023;13(5):1198. doi: 10.3390/life13051198. 37240843 PMC10221969

[29] Ostermann M, Joannidis M, Pani A, et al. Patient selection and timing of continuous renal replacement therapy. Blood Purif. 2016;42(3):224–237. doi: 10.1159/000448506. 27561956

[30] Koyner JL, Carey KA, Edelson DP, et al. The development of a machine learning inpatient acute kidney injury prediction model. Crit Care Med. 2018;46(7):1070–1077. doi: 10.1097/CCM.0000000000003123. 29596073

