# Supplementary material for: Implementation quality as a modifiable determinant of filter lifespan in regional citrate anticoagulation: a real-world clustering-adjusted study
Source: Ren Fail. 2026 Apr 22;48(1):2656550. doi: 10.1080/0886022X.2026.2656550 (PMC13103979; doi:10.1080/0886022X.2026.2656550)
Supplement: Supplemental Material [file IRNF_A_2656550_SM4453.docx]

Table S1. Full mixed-effects Cox model results for filter clotting.

| **Variable** | **aHR** | **95% CI** | ***p* - value** |
| --- | --- | --- | --- |
| Anticoagulant (versus RCA) | - | - | - |
| Heparin | **1.78** | 1.14–2.77 | 0.011 |
| Nafamostat | 1.31 | 0.78–2.19 | 0.31 |
| Age (per 10 years) | 1.02 | 0.89–1.17 | 0.76 |
| Mechanical ventilation | 1.24 | 0.82–1.87 | 0.31 |
| CRRT mode (versus CVVH) | - | - | - |
| CVVHD | 0.91 | 0.54–1.53 | 0.72 |
| CVVHDF | 1.08 | 0.68–1.72 | 0.75 |
| Blood flow rate (per 10 mL/min) | 0.96 | 0.89–1.04 | 0.32 |
| Machine type (versus Fresenius) | 1.18 | 0.77–1.81 | 0.45 |
| Sepsis | 1.14 | 0.76–1.71 | 0.52 |
| Liver failure | 1.31 | 0.72–2.38 | 0.38 |

Table S2. Univariate and multivariable logistic regression for filter clotting within the RCA group.

| **Variable** | **Univariate OR (95% CI)** | ***p* - value** | **Multivariable OR (95% CI)** | ***p* - value** |
| --- | --- | --- | --- | --- |
| No timely monitoring (versus timely) | 2.26 (1.32–3.87) | 0.003 | 2.18 (1.24–3.84) | 0.007 |
| Time to first monitoring (per h) | 1.16 (1.03–1.31) | 0.01 | 1.15 (1.02–1.30) | 0.02 |
| Blood flow rate (per 10 mL/min) | 0.94 (0.85–1.04) | 0.22 | 0.95 (0.86–1.05) | 0.31 |
| CRRT mode (versus CVVH) | - | - | - | - |
| CVVHD | 0.83 (0.38–1.81) | 0.64 | 0.79 (0.35–1.78) | 0.57 |
| CVVHDF | 1.21 (0.61–2.40) | 0.58 | 1.18 (0.58–2.40) | 0.65 |
